# Soil and Crop Management Practices to Minimize the Impact of Waterlogging on Crop Productivity

**DOI:** 10.3389/fpls.2019.00140

**Published:** 2019-02-12

**Authors:** S. M. Nuruzzaman Manik, Georgina Pengilley, Geoffrey Dean, Brian Field, Sergey Shabala, Meixue Zhou

**Affiliations:** ^1^Tasmanian Institute of Agriculture, University of Tasmania, Prospect, TAS, Australia; ^2^Hubei Collaborative Innovation Center for Grain Industry/School of Agriculture, Yangtze University, Jingzhou, China

**Keywords:** agronomic practices, soil engineering, drainage, genetic solutions, waterlogging tolerance

## Abstract

Waterlogging remains a significant constraint to cereal production across the globe in areas with high rainfall and/or poor drainage. Improving tolerance of plants to waterlogging is the most economical way of tackling the problem. However, under severe waterlogging combined agronomic, engineering and genetic solutions will be more effective. A wide range of agronomic and engineering solutions are currently being used by grain growers to reduce losses from waterlogging. In this scoping study, we reviewed the effects of waterlogging on plant growth, and advantages and disadvantages of various agronomic and engineering solutions which are used to mitigate waterlogging damage. Further research should be focused on: cost/benefit analyses of different drainage strategies; understanding the mechanisms of nutrient loss during waterlogging and quantifying the benefits of nutrient application; increasing soil profile de-watering through soil improvement and agronomic strategies; revealing specificity of the interaction between different management practices and environment as well as among management practices; and more importantly, combined genetic, agronomic and engineering strategies for varying environments.

## Introduction

Waterlogging is one of the focal abiotic stresses, which affects crop growth (Linkemer et al., 1998; Setter and Waters, 2003; Lone et al., 2018). It has become the key constraint to crop production in the temperate high rainfall zone (HRZ) of Australia (Acuña et al., 2011), particularly in regions with duplex soils (Yaduvanshi et al., 2012). Global climate change causes waterlogging events to be more frequent, severe, and unpredictable (Jackson and Colmer, 2005; Intergovernmental Panel on Climate Change [IPCC], 2014). Some currently wet areas will become wetter and prolonged waterlogging will also become more prevalent (Dore, 2005; Intergovernmental Panel on Climate Change [IPCC], 2014). The value of this loss is also signficiant to the Australian grains industry where waterlogging causes an estimated annual production loss of AU$180 M (Pang et al., 2004) with a greater proportion of this being incurred in Western Australia (Zhang et al., 2006). Waterlogging caused 40–50% wheat yield reduction in a wet year (Zhou, 2010) resulting in AU$100 M in crop losses (Zhang et al., 2004).

Waterlogging is also a matter of worldwide concern affecting 16% of the soils in the United States, 10% of the agricultural lands of Russia and irrigated crop production areas of India, Pakistan, Bangladesh, and China (Yaduvanshi et al., 2014; Food and Agriculture Organization [FAO], 2015). Globally, between 10 and 15 million ha of wheat are affected by waterlogging annually causing yield losses of between 20 and 50% (Hossain and Uddin, 2011). Waterlogging also causes yield losses in other grain crops such as barley, canola, lupins, field peas (Bakker et al., 2007; Romina et al., 2018), lentils and chickpeas (Solaiman et al., 2007).

Appropriate soil and crop management practices improve soil quality and crop productivity, through improved ecological and economical flexibility by reducing the need for additional agricultural land (Setter and Belford, 1990; Tilman et al., 2002; Shaxson and Barber, 2003). Improved soil management can increase infiltration, reduce surface runoff, and additionally improve availability of water and nutrients to plants (Amare et al., 2013; Negusse et al., 2013; Schmidt and Zemadim, 2015; Masunaga and Marques Fong, 2018). Crop management can contribute to higher yields (Soomro et al., 2009; Amare et al., 2013). This review focuses on the impact of waterlogging on soil properties, plant growth and agricultural management practices to mitigate waterlogging. The gaps in current knowledge, technology and farm practices are identified, and recommendations are made for future opportunities to ensure sustainable soil and crop management under waterlogged conditions.

## Waterlogging Effect on Soil and Plant Growth

**FIGURE 1:**
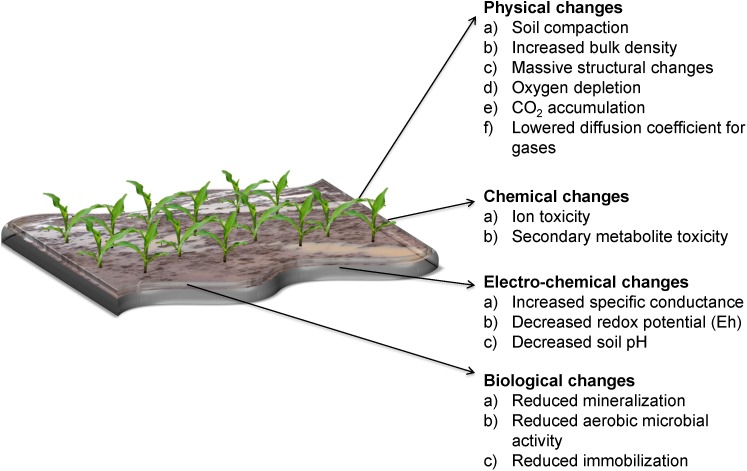
Effects of waterlogging on soil properties.

**FIGURE 2:**
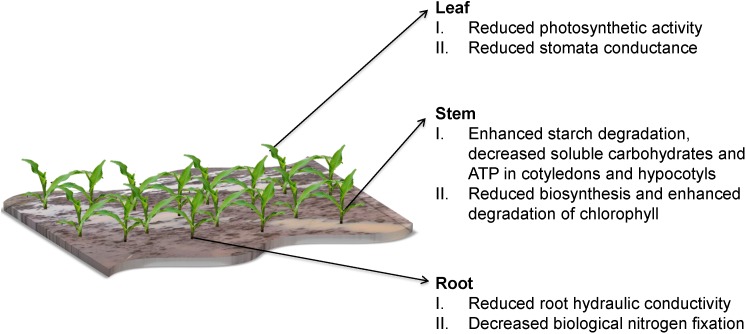
Effects of waterlogging on plant growth.

Waterlogging impedes the ability of soil to provide an optimum medium for plant growth and alters its physical, chemical, electro-chemical and biological characteristics as summarized in Figure 1 (Pulford and Tabatabai, 1988; Glinski, 2018; Ferronato et al., 2019). This has a significant impact on the development of the root biomass and subsequently on plant overall development (Figure 2) (Ernst, 1990; Pierret et al., 2007; de San Celedonio et al., 2016). Fundamentally, the soil should have optimal water and air content for the proper physiological performance of the all phases of plant growth (Crawford, 1982).

Waterlogging hinders the growth of plants by reducing the dispersal of oxygen through the pore spaces in the soil around the root zone (Drew and Sisworo, 1977; Lee T.G. et al., 2007; Christianson et al., 2010) with the dispersal of oxygen being 320K times lower than that in non-submerged soils (Armstrong, 1980; Barrett-Lennard, 2003; Lee T. et al., 2007; Colmer and Greenway, 2011). The plant root needs an adequate supply of oxygen so as to fulfill the water and nutrient requirements of the shoots, and the soil oxygen concentration should be above 10% where atmospheric molecular oxygen concentration is 21% (Sojka and Scott, 2002; Brady and Weil, 2008; Colmer and Greenway, 2011; Morales-Olmedo et al., 2015; da Ponte et al., 2019). Under waterlogging conditions, oxygen demand to the root tip and to the rhizosphere is supplied by forming aerenchyma through removal of some cells of the cortex and these remove excess gases from the root and soil (Armstrong, 1980; Colmer and Greenway, 2011). The gas exchange between soil and atmosphere in a well aerated soil is amply rapid to decelerate O_2_ deficiency and toxicity caused by excess CO_2_ or other gases such as ethylene and methane (Sojka and Scott, 2002). A number of hydroponic or inert substrate experiments assessed the effect of hypoxia and anoxia on plants in waterlogged conditions, and determined the soil is a vital factor (Morard and Silvestre, 1996; Striker, 2008; Arbona et al., 2009; Bai et al., 2013; Morales-Olmedo et al., 2015). Excessive water content in the soil upon waterlogging decreases O_2_ diffusion capacity, leading to hypoxic or even anoxic environments that inhibit the activity of nitrifying communities, resulting in depleted soil N availability that will negatively affect N-dependent crop productivity (Jaiswal and Srivastava, 2018; Nguyen L.T. et al., 2018). Thus, the rate of nitrification is estimated to decrease in response to waterlogging conditions (Reddy and Patrick, 1975; Laanbroek, 1990).

Moreover, decreasing molecular oxygen prompts a sequence of changes in the physico-chemical properties of the soil (Ponnamperuma, 1984). Many of these also change soil chemical and electro chemicals by decreasing redox potential and excess electron changes, such as Fe^3+^ and Mn^4+^ to Fe^2+^ and Mn^2+^, correspondingly (Ponnamperuma, 1984; Jackson and Colmer, 2005; Singh and Setter, 2017). Thus, solubility of iron and manganese rises to toxic levels, which are potentially damaging to plant roots (Jones and Etherington, 1970; Aldana et al., 2014; Marashi, 2018; Sharma et al., 2018). Apart from the elemental toxicities to the sensitive root tips, increased concentration of secondary metabolites such as phenolics and volatile fatty acids may become injurious in the low-pH rhizosphere (Pang et al., 2007a; Shabala, 2011; Coutinho et al., 2018). pH values of waterlogged soil can be further reduced by the accumulation of volatile organic acids as well as the high concentration of CO_2_ (Greenway et al., 2006) which reduces root growth (Boru et al., 2003). As mentioned, another potential toxic metabolite found in waterlogged soil is ethylene, which suppresses root expansion growth (Drew and Lynch, 1980; Shabala, 2011). In addition, with the re-introduction of oxygen at the recovery phase, the remaining ethanol in anoxic cells will be transformed into acetaldehyde which may cause cell injuries (Bailey-Serres and Voesenek, 2008). Under abiotic stress conditions, reactive oxygen species (ROS) levels are always elevated compared to pre-stress levels (Miller et al., 2008). Excessive production of various ROS such as superoxide radicals, hydroxyl radicals, hydrogen peroxide, and singlet oxygen found in hypoxia-stressed leaf and root tissues (Blokhina et al., 2003; Sairam et al., 2009; Petrov et al., 2015; Shabala et al., 2016) can also cause severe damage to plants. All of these lead to restricted root growth, reduced tiller number, premature leaf senescence and production of sterile florets thus affecting the grain yield (Collaku and Harrison, 2005; Hossain and Uddin, 2011; Cannarozzi et al., 2018).

Even though the accumulation of phytotoxic compounds requires time, the absence of oxygen alone is enough to change the plant metabolic activities to critical levels (Geigenberger, 2003; Perata et al., 2011). O_2_ deficiency during waterlogging leads to reduced availability of energy in the roots (Armstrong et al., 1991) and, as a result, energy-dependent processes such as nutrient uptake are inhibited (Setter and Belford, 1990). N deficiency is believed to be the other cause of the suppression of growth under waterlogging (Cannell et al., 1980; Robertson et al., 2009; Wollmer et al., 2018). Carbohydrate (the energy reserve) production reduced dramatically during complete submergence or subsequent de-submergence due to reduced photosynthetic rate (Sarkar et al., 2006; Pérez-Jiménez et al., 2018), reduced stomatal conductance, declined root hydraulic conductivity and reduced translocation of photo assimilates (Parent et al., 2008). One of the first plant responses to waterlogging is the reduction in stomata conductance (Folzer et al., 2006), for example, fast stomata closure in barley (Yordanova et al., 2005) and pea (Zhang and Zhang, 1994). The stomata closure was attributed to abscisic acid (ABA) transport from older to younger leaves or *de novo* synthesis of this hormone (Zhang and Zhang, 1994). Waterlogging also decreases the leaf chlorophyll content (Malik et al., 2001; Ashraf et al., 2011; Li et al., 2018; Ma et al., 2018). This decrease in chlorophyll directly or indirectly affects the photosynthetic capacity of plants (Azhar et al., 2018; Rasheed et al., 2018; Yu et al., 2019). This decrease in transpiration and photosynthesis is attributed to stomata closure (Ashraf and Arfan, 2005; Tian et al., 2018) which restricts CO_2_ movement (Jackson and Hall, 1987; Malik et al., 2001; Chu et al., 2018). To summarize, waterlogging affects overall plant growth, which leads to a substantial yield loss (Kumar, 2018; Romina et al., 2018; Wollmer et al., 2018).

## Soil Management

Soil management practices such as drainage, tillage and traffic control can alter soil structure directly or indirectly (Pagliai et al., 2004; Unger et al., 2018). Many of these changes are relatively short term and reversible. Management-induced changes in the quantity and characteristics of soil can also lead to changes in soil structure that are much more persistent. Management practices which are sustainable must maintain the structure of soil, over the long term, in a state that is optimum for a range of processes related to crop production and environmental quality (Bogunovic et al., 2017; Belmonte et al., 2018).

Soil surface biological communities provide critical functions in many ecosystems by controlling infiltration and thus ensure suitable water availability for crop, soil biota, nutrient cycles and vascular vegetation (Chamizo et al., 2016). They increase biodiversity, accelerate soil formation rates, and contribute to the biogeochemical cycling of nutrients by fixing atmospheric carbon (C) and nitrogen (N) (Elbert et al., 2012; Weber and Hill, 2016). Therefore, a key consideration in designing management practices must be targeting the soil surface.

Various soil management practices can mitigate adverse effects of waterlogging stresses. Here, we review some soil management practices emphasizing the system used for waterlogging-prone areas.

### Controlled Traffic Farming (CTF)

**FIGURE 3:**
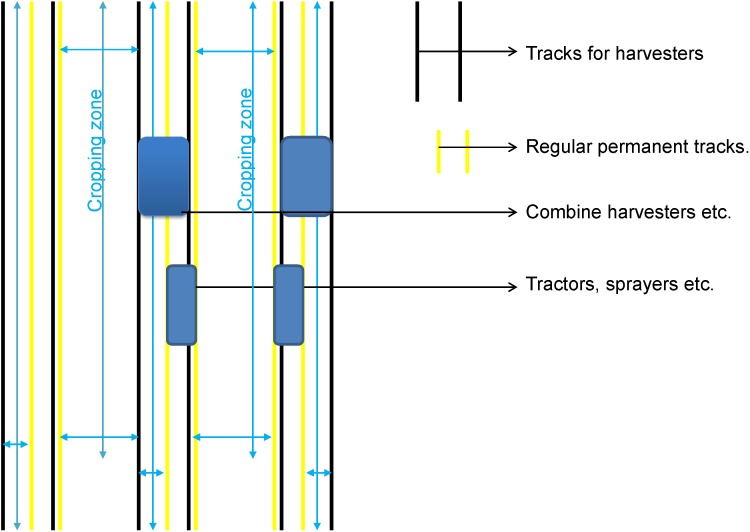
Controlled traffic farming.

Controlled traffic farming (CTF) is a management system to control extensive unsystematic trafficking by farm machinery/vehicles and protect soil structure from indiscriminate change (Hamza and Anderson, 2005). CTF (Figure 3) is a crop production system where the crop region and traffic-lanes are markedly divided (Taylor, 1983; Raper, 2005; Bochtis and Vougioukas, 2008). It creates two distinct zones, the crop region which is non-trafficked and traffic region or non-cropped. Consequently, CTF systems always maintain the crop region unaffected by wheel tracks, whereas the traffic zone develops into a compacted zone for machinery draught efficacy (Taylor, 1992). CTF is differentiated from conventional traffic practices, known as random traffic farming (RTF) by reducing the trafficked area.

Random traffic farming or disorganized traffic causes increases in the soil bulk density resulting in increases in strength limiting soil porosity further leading to soil compaction (Tullberg, 2000; Chen et al., 2010; Rasaily et al., 2012). RTF has an adverse effect on a wide range of soil physical characteristics, including the infiltration and drainage of water, amenability for crop sowing, establishment and nurture, and soil gaseous exchange medium and soil-living organism (Gasso et al., 2013; Gasso et al., 2014). Due to random traffic a large amount of soil is adversely affected resulting in soil degradation issues in Australia (4 million ha), Europe (33 million ha), Asia (10 million ha), Africa (18 million ha) (Flowers and Lal, 1998; Hamza and Anderson, 2003; Shahrayini et al., 2018).

The advantages of CTF have been well documented. These include reduced incidence of waterlogging, soil compaction, erosion, tillage, water and nutrient losses and thus increased crop yield (Morling, 1982; Raper, 2005; Tullberg et al., 2007; Chamen, 2015). Adoption of CTF by Australian grain growers was 3% in 2003 (Price, 2004), 15% in 2006 and 36% in 2008 (Robertson, 2008). In Australia a minimum of 10% yield improvement in barley, wheat, and canola across diverse soil types has been noted along with decreases in fuel consumption of machinery due to improved draught on the traffic area (Webb et al., 2004; Li et al., 2007; Robertson et al., 2007; Lorimer, 2008; Davies et al., 2012). In Europe yield of cereal crops (i.e., wheat and barley) increased from 9 to 21% with CTF compared with RTF (Gasso et al., 2013). Similarly, wheat yield with CTF increased by 6.9% compared to traditional tillage in China (Qingjie et al., 2009).

### Strategic Deep Tillage and Subsoil Manuring

Strategic deep tillage (SDT) is a single or occasional practice with a deep ripper, rotary, spader, mouldboard plow or disk plow to help sustain the long-standing productivity of the no-till system (Renton and Flower, 2015; Roper et al., 2015; Rincon-Florez et al., 2016; Kuhwald et al., 2017; Scanlan and Davies, 2019). Deep tillage or soil cultivation to loosen compact soil layers, particularly the clay subsoil, has been suggested to improve drainage in the subsoil, thus reducing waterlogging (Gardner et al., 1992). The technique may also incorporate slotting of gypsum to reduce sodicity and improve soil structure, which also reduces waterlogging (Crabtree, 1989; McFarlane and Cox, 1992).

Deep ripping loosens hard layers of soil by using sturdy tines to 35–50 cm depth. It is not suitable for all soils and crops, therefore season, timing, soil type, tine spacing, shallow leading tines, soil moisture content, and working depth are all factors that need to be taken into consideration. The benefit of combined CTF and SDT may last for three seasons but can be as long as ten seasons with average wheat yield increase in Western Australia being 0.6 t/ha and 0.5 t/ha at 12 sites and 16 sites, respectively (Davies et al., 2012; Roper et al., 2015). Schneider et al. (2017) conducted a meta-analysis of 1530 yield comparison at 67 experimental sites across Germany, United States, Canada, and India and showed increased yield of greater than 6% between deep and ordinary tillage systems.

There are, however, several disadvantages of deep ripping including its short-term nature (especially if traffic is not managed to reduce re-compaction), effectiveness in hostile sub-soils, such as acidity, sodicity or subsoil salinity, and its implementation on a large scale (Bakker et al., 2007). In this case, amelioration with gypsum or lime may be helpful to stabilize the soil (Henry et al., 2018; Matosic et al., 2018). Although yield benefit from SDT has been demonstrated, organic matter, texture and soil nutrients distribution within the root zone need appropriate long term agronomic management to maximize the benefit (Roper et al., 2015).

Another way of reducing waterlogging is through a similar practice where large volume of organic matter with high N levels are placed within and above the heavy clay layers. This practice is referred to as sub-soil manuring (Gill et al., 2009; Peries, 2013; Celestina et al., 2018). In south eastern Australia approximately 80% of the cropping zone of medium annual rainfall (375–500 mm) and high rainfall (>500 mm) are affected by subsoil constraints (Grains Research, and Development Corporation [GRDC], 2016). Amelioration of subsoil constraints may be possible by slotting large quantities (>10 t/ha) of organic matter and other amendments (Sale, 2014; Armstrong et al., 2015). Experiments carried out in Victoria, Australia demonstrated that lucerne pellets and commercial poultry manure can significantly improve soil properties and crop growth as well as yield by improving subsoil structure and supplying N (Gill et al., 2009, 2012).

The increase in water extraction by roots also provides a greater buffer for subsequent waterlogging events. However, adoption on a commercial scale has remained low due to a combination of high upfront costs (up to $1200/ha) to implement, variable yield responses and logistical constraints such as the limited availability of suitable organic matter sources and access to appropriate machinery (Nicholson, 2016; Armstrong et al., 2017).

### Drainage Systems

Land drainage is one of the main approaches to improve yields per unit of accessible agricultural area (Bos and Boers, 1994; Malano and van Hofwegen, 2006; Singh, 2018b). Reducing soil submergence, salinity control, and making new land accessible for agriculture are the three main objectives of agricultural drainage (Singh and Panda, 2012; Singh, 2018b). However, drainage, an efficient agriculture engineering system to combat waterlogging, has not been given equal importance when compared to irrigation by individual farmers and governmental agencies.

Drainage is used to alleviate waterlogging not only in some parts of Australia (Cox et al., 1994; Milroy et al., 2009), but also world-wide. Various studies, conducted in England, Europe, and North America indicate that drainage can effectively lower the water table and improve crop yields (Cannell and Jackson, 1981; Evans and Fausey, 1999; Bullock and Acreman, 2003; Blann et al., 2009; Smedema et al., 2014; Gramlich et al., 2018). It was also reported to greatly reduce wheat yield losses due to waterlogging in south-western Victoria (Gardner and Flood, 1993; Christy et al., 2015; Feng et al., 2018). Despite the yield losses associated with waterlogging on prone Australian texture-contrast soils, large scale adoption of drainage is still limited in the HRZ (Cox et al., 2005; Rengasamy, 2006; Christy et al., 2015). Various methods have been recommended to mitigate the waterlogging problems, such as surface drainage, subsurface drainage, and mole drains (Muirhead et al., 1996; Misak et al., 1997; Konukcu et al., 2006; Ram et al., 2007; Ritzema et al., 2008; Kazmi et al., 2012; Singh, 2012, 2016; Singh and Panda, 2012).

### Surface Drainage

Surface drainage is defined as the safe removal of excess water through constructed channels from the land surface (Ritzema et al., 2008; Ayars and Evans, 2015). Surface drains such as ‘spoon-drains,’ ‘W-drains’ and reverse seepage interceptor banks and interceptor drains have been used to alleviate the conditions of waterlogging in south-western Australia (Cox et al., 1994). Surface drainage systems have been shown to be cost effective with cost-benefit ratios being in the range from 1.2 to 3.2, internal return rates from 20 to 58%, and payback periods from 3 to 9 years (Ritzema et al., 2008). The simplest and cheapest option is to maintain existing surface drains and install extra drains along fence lines or through depressions considering adequate size and proper position. Preventing water flow from upper to lower paddocks with cut-off drains should also be implemented (Palla et al., 2018). However, the success of surface drainage is often limited due to the poor lateral water movement or internal soil drainage properties, which results in poor drainage in the vicinity of the drains (McFarlane and Cox, 1992; Cox and McFarlane, 1995; Saadat et al., 2018). Both surface and subsurface drainage may thus be required to solve these problems.

### Raised Bed System

**FIGURE 4:**
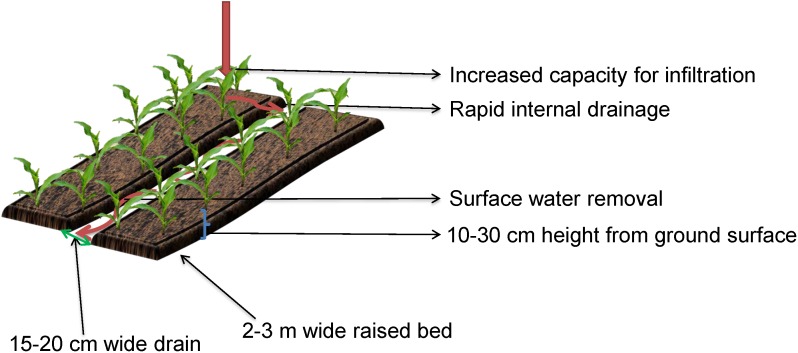
Raised bed cropping system.

The use of raised beds (Figure 4) is an important soil management option to improve crop yield, soil structure, and productivity under waterlogged conditions (Hamilton et al., 2000; Bakker et al., 2005b; Hussain et al., 2018). The beds reduce waterlogging and improve the overall soil structure through installing shallow drains or furrows approximately 15–20 cm wide at regular intervals. These are then used for tractors, and sprayers to control traffic movement over the paddock (control traffic farming) (Collis, 2015). The 2–3 m wide and 10–30 cm height bed is formed using soil creating a raised bed allowing water to drain away from the plant root zone and reducing the likelihood of waterlogging damage (Riffkin et al., 2003; Bakker et al., 2007; Gibson, 2014; Ghazouani et al., 2015). Planting on beds also diminishes pesticide applications due to a reduction in fungal and other diseases with improved radiation interception, acquisitive temperature and reduced humidity in the canopy (Alwang et al., 2018).

When seasonal conditions are appropriate, raised beds can significantly increase grain yield under waterlogged conditions compared with crops grown conventionally on flat ground (Bakker et al., 2007; Acuña et al., 2011). In Australia, raised beds are used in irrigated agriculture in New South Wales and north-west Victoria, as well as in cotton growing areas in New South Wales to minimize the impacts of waterlogging (Bakker et al., 2005a; Bakker et al., 2007). The use of raised beds is also prominent in high rainfall areas across Victoria and experiments demonstrated that wheat and barley yield increased by 50% and 30%, respectively (Collis, 2015), and were proposed for the waterlogged duplex soils of Western Australia (Bakker et al., 2005a; Bakker et al., 2005b), and for the frequently waterlogged arable land across the south-eastern wheat belt of Western Australia (Bakker et al., 2007). Permanent raised beds and furrow systems are also used to manage waterlogging in Mexico (Roth et al., 2005) and coastal lowlands in humid tropical regions in some South Asian countries (Velmurugan et al., 2016), consistently delivering higher returns based on cost–benefit analyses.

While raised beds have had a positive impact on alleviating the effects of waterlogging they also have a number of disadvantages. These include the cost of adapting and modifying machinery, greater difficulty in controlling sowing depth and seed placement on beds, management of drainage water, limited use where the water table is too high, stubble handling and fodder conservation, firefighting and mustering livestock, the possibility of pesticide contamination into waterways and leaching into the water table and inefficiencies of machinery and weed control in furrows (Bakker et al., 2005b; Gibson, 2014). When raised beds were compared with hump and hollow surface drainage in waterlogged pastures at Derrinallum, Victoria it was concluded that the use of raised beds for the growing of pastures for grazing had little to offer the sheep industry (Ward et al., 2007). This poses a significant research question around suitability of raised beds in the many mixed farming systems that operate across the HRZ of Australia.

### Subsurface Drainage

Poor subsurface water movement occurs due to the inability of water to move through soil as a result of heavy soil texture, compacted layers and naturally created or induced hard pans as well as water moving downhill from upper slopes or from springs, raising the water table (Ward et al., 2018). Subsurface drainage lowers the water table or perched water and ensures a suitable environment in the root region where waterlogging occurs (Christen et al., 2001; Xian et al., 2017). About 50% of waterlogged areas in western Europe, 20–35% of total cultivated land in Europe and North America, 5–10% in Asia, Australia, and South America, and 0–3% in Africa have used subsurface drainage measures (Food and Agriculture Organization [FAO], 2002). Subsurface drainage systems consist of open and pipe drains with variable drain depth and spacing (Ritzema et al., 2008). The systems are more effective in areas where the subsoils are sufficiently stable (Gardner et al., 1992) and not exhibiting characteristics of hostile sub-soils such as sodicity.

Subsurface pipe drains are the main forms of subsurface drainage found in the HRZ of Australia (Christen et al., 2001). Usually, the type of drain to be installed depends on topography, soil characteristics and rate of drainage required. There has been successful use of sub-surface drainage in areas of Tasmania, Australia and grain growers are willing to invest in drainage as a long-term solution to waterlogging (Gibson, 2014). This is also supported by a study into the economics of drainage, which indicated that subsurface drainage provided crop growers with the confidence to target high potential yields where the cost benefit was positive (Bastick and Walker, 2000).

Although, subsurface deep drains (depth > 1.75 m) are recommended in India (Gupta, 2002), these deep drains can be economically installed only by mechanical construction practices, and the deeper the drain the higher the installation cost (Gupta, 1997). In some parts of Australia, several types of subsurface drainage were found to be unsuccessful because they were expensive and failed to control surface water (McFarlane and Cox, 1992). Managing waterlogging with horizontal tile drainage systems (using a combined drainage system with tube wells plus horizontal drainage systems) is more beneficial in maintaining the water table within the desirable depths (Chandio et al., 2013). In Australia, subsurface drainage such as tile and mole drainage are shown to be particularly useful for irrigated high-value crops such as perennial horticulture, cotton, pasture, sugarcane and perennial pastures for dairying (MacEwan et al., 1992; Christen et al., 2001).

### Subsurface Pipe Drains

**FIGURE 5:**
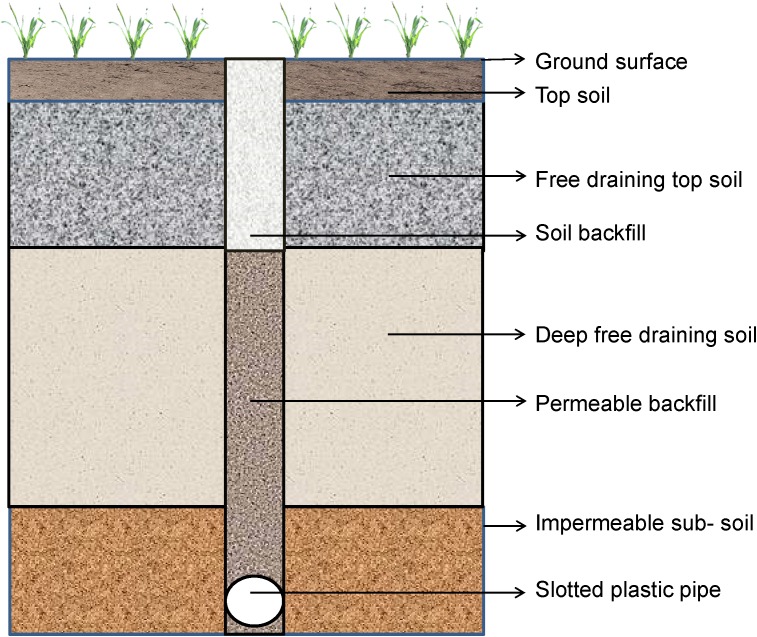
Horizontal drainage system.

Horizontal subsurface (Figure 5) drainage removes excess water from the crop root zone (Tanji, 1990; Teixeira et al., 2018). Below the ground surface, the drainage structure comprises a grid of perforated pipes connected to control the water table. Tile drainage is a form of horizontal subsurface drainage consisting of small pipes of concrete or burnt clay installed at a certain depth below the ground surface (King et al., 2014). Tile drainage is used widely in agricultural areas where subsoil surplus water is a common problem (Williams et al., 2015). To improve the system gravel is usually used above the tile drains as a backfill material in the areas where there is shallow groundwater and heavy soil conditions (Filipović et al., 2014).

Besides water table control, horizontal drainage controls soil salinity in the root zone of the soil by leaching out the concentrated and harmful salt solutions (Christen and Skehan, 2001). This is an established and significantly relevant system for saline land reclamation in Australia and India in irrigation areas where excess soil salinity is the prime limitation in agricultural production (Christen and Skehan, 2001; Prathapar et al., 2018). However, this method may not be suitable for agricultural lands where the top soils are prone to seasonal waterlogging due to poor hydraulic conductivity and the need to find appropriate outfall for drained water (Christen and Skehan, 2001; Food and Agriculture Organization [FAO], 2002; Prathapar et al., 2018; Singh, 2018a).

### Vertical Subsurface Drainage

**FIGURE 6:**
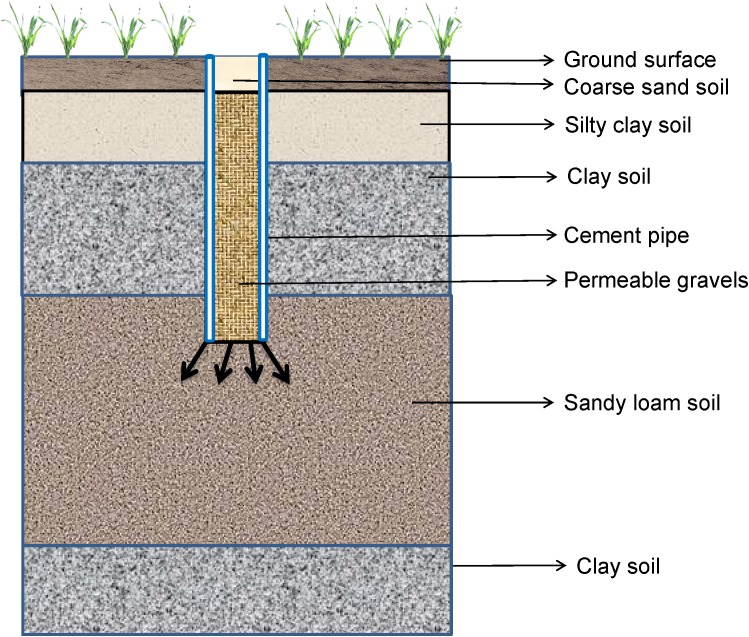
Vertical drainage system.

Vertical drainage (VD) (Figure 6) is used for controlling rising groundwater levels in some parts of Australia such as Burdekin, Kerang, and Shepparton (Christen et al., 2001; Kijne, 2006). Recent results showed that installing VD can reduce the duration of seasonal waterlogging in Bihar, India (Prathapar et al., 2018). Various types of vertical drains have been used to consolidate the soil, such as prefabricated vertical drains (PVDs), sand compaction piles, sand drains, gravel piles and stone columns (Indraratna et al., 2005; Indraratna, 2017). Recently, PVDs have been installed in Brisbane and Ballina in Australia (Indraratna, 2017). The VD system has some advantages over other subsurface drainage systems. For example, VDs are often preferred because of relative low capital cost and the length of open surface drains is less with VD when compared with other types of drainage (Christen et al., 2001). VDs also allow the groundwater level to be lowered to a greater depth than other drainage systems (Kruseman and Ridder, 1990). However, the maintenance and operational costs are higher than horizontal drainage systems as it involves high energy to operate a network of tube wells (Christen et al., 2001; Food and Agriculture Organization [FAO], 2002; Prathapar et al., 2018). The effectiveness of the VD system is demonstrated by the drop in the groundwater level, therefore, the system is more suitable for an area with fluctuating high levels of groundwater.

### Mole Drains

**FIGURE 7:**
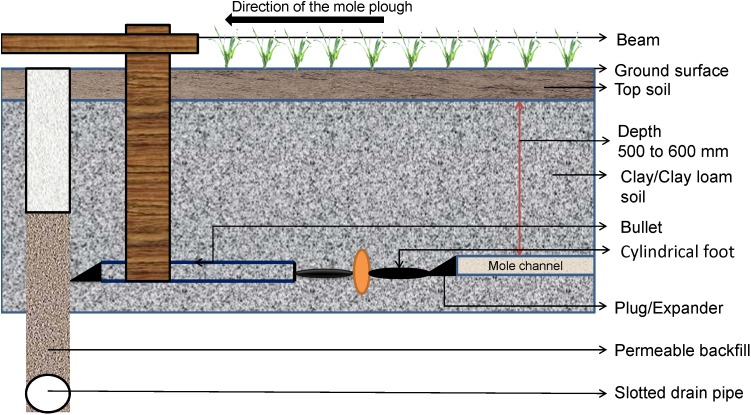
Mole drainage system.

Mole drainage (Figure 7) is another form of subsurface drainage. Its effects on reducing waterlogging have been shown in Victoria, Australia (Frank, 2010; Gibson, 2014). Mole drain systems were found to improve performance in terms of growth parameters, yield attributes and economic parameters of soybean (*Glycine max*) and wheat (*Triticum aestivum*) in Madhya Pradesh, India (Dhakad et al., 2018).

Mole drains are a semi-permanent system from a layout and operational point of view and are similar to tile drainage. Although costing less than tile drainage, they do require more maintenance (Tuohy et al., 2016, 2018; Dhakad et al., 2018). This drainage system is generally installed to manage rising groundwater levels and land salinization problems (Robinson et al., 1987; Castanheira and Serralheiro, 2010; Kolekar et al., 2014). Mole drainage relies on closely spaced channels and subsoil cracks to quickly send surplus soil water to the tile or agricultural (ag) pipe drainage system throughout the season (Childs, 1943; Hallard and Armstrong, 1992; Tuohy et al., 2015, 2016). Mole drains are installed in close proximity to tile drains and are most suitable for low-permeability heavy soils such as clay (Monaghan et al., 2002; Monaghan and Smith, 2004). These drains should be installed at less than 600 mm from the ground surface and form 40–50 mm diameter circle of drainage (Gibson, 2014). A mole drain can be formed by dragging a metal object (viz. a blade like bullet with cylindrical foot, or mole plow) through the soil which creates an open channel. The installation cost of mole drainage is low but the moles should be re-formed at approximately 2- to 5-year intervals to uphold the channel integrity and optimize overall performance of the system (Tuohy et al., 2016, 2018). Combined drainage systems (mole and tile drainage) can be used efficiently to simulate water balance and drainage network system over a watershed, and to aid drainage management in a floodplain landscape (Tuohy et al., 2018).

## Crop Management

There are a large and diverse number of crop management practices used by grain growers to alleviate the effects of waterlogging. These include: crop choice, waterlogging tolerant crop varieties, bio-drainage, and different agronomic practices such as sowing time, nutrient application and plant growth regulators (PGRs).

### Early Sowing and Vigorous Crops

Crop management options to increase crop water use and decrease the incidence of waterlogging include early sowing and higher sowing rates (Gardner et al., 1992). Early sowing of wheat varieties showed better performance (Setter and Waters, 2003; Bassu et al., 2009; Ali et al., 2018) due to reduced risk of waterlogging damage through de-watering of the soil profile and avoiding waterlogging at vulnerable early growth stages (Gardner and Flood, 1993). Wheat, barley and rapeseed plants were less affected by early waterlogging (vegetative stages) than late (reproductive stages) (Ploschuk et al., 2018; Wollmer et al., 2018). Early sowing can also avoid late season terminal waterlogging events (Stapper and Harris, 1989). In addition, higher sowing rates can compensate for reduced tiller numbers and fertile heads (Watson et al., 1976; Belford et al., 1992).

Early crop vigor can be another important trait for waterlogging tolerance in the field (Sundgren et al., 2018). Tillering and reproductive stages are crucial for waterlogging tolerance in crops such as wheat and barley (Setter and Waters, 2003). Reduced nitrogen uptake is one of the main effects of waterlogging stress in crops (Jaiswal and Srivastava, 2018; Nguyen L.T. et al., 2018). Early vigor may be linked with increased uptake of nitrogen (Liao et al., 2004; Sundgren et al., 2018). However, under normal conditions seedling growth rates can also vary with genotypic differences (Rebetzke et al., 2004). Further research may provide more insight into the interactions and possible use of early vigor to mitigate the effects of waterlogging.

### Bio-Drainage

The incorporation of herbaceous perennial legumes such as lucerne, clovers and Messina (*Melilotus siculus*) adapted to waterlogging and inundation into cropping systems has been suggested to reduce waterlogging (Cocks, 2001; Nichols, 2018). Usually these deep rooted pasture plants can extract water and dry the soil to greater depths than most annual crops (McCaskill and Kearney, 2016). However, there is significant variation in tolerance to waterlogging between different pasture species (Cocks, 2001), and their suitability for grain production systems and how they would be integrated to provide maximum benefit has been identified as a gap in knowledge and warrants further investigation.

Bio-drainage or bio-pumping is the VD of soil water using specific types of fast growing tree vegetation with high evapotranspiration demand and is considered an economically viable option in dealing with the drainage congestion and environment hazards (Kapoor, 2000; Heuperman and Kapoor, 2003; Dash et al., 2005; Sarkar et al., 2018; Singh and Lal, 2018). Bio-drainage vegetation has been demonstrated to lower the rising water table around the root zone of adjacent cultivated crops in waterlogged areas through drainage (Roy Chowdhury et al., 2011; Sarkar et al., 2018; Singh and Lal, 2018).

Lowering of the rising water table is apparent within 5–10 years of growing vegetation and trees (Silberstein et al., 1999; Singh and Lal, 2018). If trees tolerant to waterlogging are introduced into the prone areas, these can easily assist in controlling water stagnation and rising water table (Banik et al., 2018; Sarkar et al., 2018). The right choice of plant species with optimum plant population and suitable plant geometry will help to control the elevated groundwater table in waterlogged areas and thus maintain the desired soil moisture regime for timely cultivation (Sarkar et al., 2018; Singh and Lal, 2018).

Prevention and remediation are the two stages of bio-drainage where the trees planted could provide a benefit to agriculture as well as resolving other issues such as waterlogging, salinity and shelter. Therefore, incorporation of a bio-drainage system with a conventional agriculture farming system could improve land and water productivity as well as the environment (Roy Chowdhury et al., 2011). Integration of bio-drainage with conventional drainage measures is an option to consider with the possibility of integration of silviculture and aquaculture with conventional agriculture to improve land and water productivity (Roy Chowdhury et al., 2011).

**FIGURE 8:**
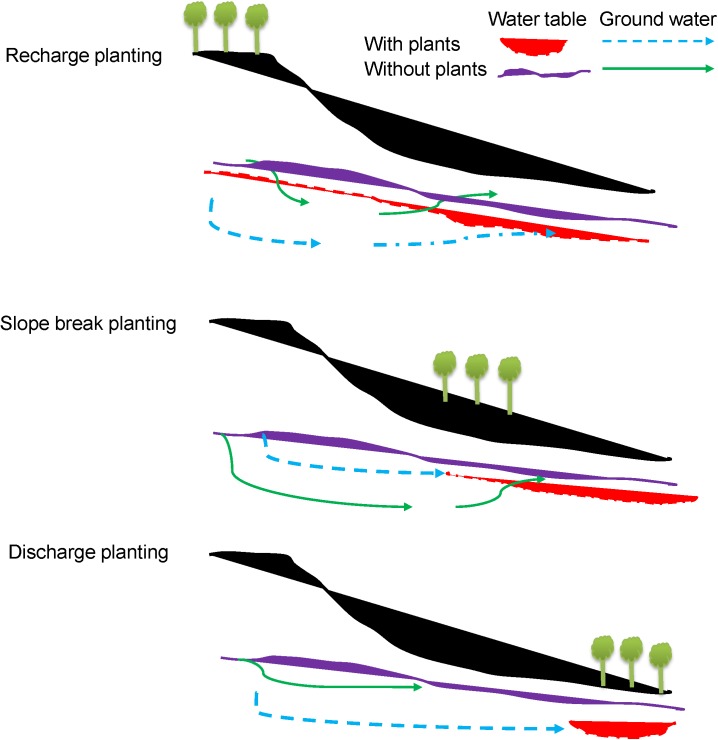
Bio-drainage system.

**FIGURE 9:**
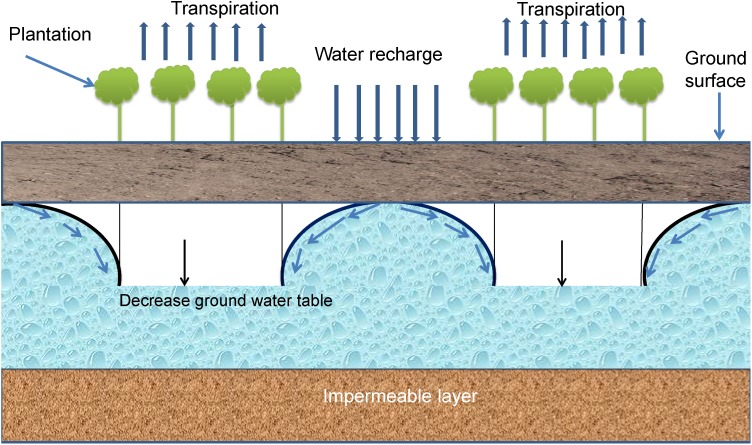
Bio-drainage planting system (modified from Donnan, 1947).

Bio-drainage systems may be established under both rainfed and irrigated conditions (Heuperman, 2000). When established under rainfed conditions, the plant roots reduce the soil bulk density and enhance groundwater recharge capacity. The roots also draw subsurface flow to reduce the water load. It is particularly useful when there is a perched water table and the water cannot easily move down the soil profile due to the presence of an impermeable layer. Recharge planting and slope break planting (Figure 8) may be adopted in the above situations. In irrigated and low lands, which are prone to waterlogging, the discharge planting method (Figure 8, 9) is useful (Donnan, 1947; Dash et al., 2005). In HRZs, application of vegetative buffer strips is also effective for controlling runoff quantity and quality (Borin et al., 2010; Kavian et al., 2018; Saleh et al., 2018). Vegetative buffer strips have also been proposed as one of the best management or conservation practices to protect water bodies from nutrients, antibiotics, bacteria and pesticides applied on adjacent agricultural fields (Muñoz-Carpena et al., 2010; Lin et al., 2011; Lerch et al., 2017; Muñoz-Carpena et al., 2018). Tree species with high transpiration rates are selected to mitigate waterlogging from canal seepage in irrigated areas. Water quality in supply canals is suitable and can be effectively intercepted and used by the trees planted along the canals (Dash et al., 2005; Singh and Lal, 2018). However, the efficiency of bio-drainage plantations needs to be verified in HRZs where permanent stagnant water is a real problem. Lack of proper knowledge, plantation techniques, expertise, motivation as well as maintenance are issues that need to be addressed to derive the real benefit of this system. In addition, the land under bio-drainage cannot be utilized for growing other crops, as in the case of conventional drainage (Dash et al., 2005; Sarkar et al., 2018; Singh and Lal, 2018). Therefore, an economic analysis of the bio-drainage endeavor is required on a case by case basis.

### Nutrient Application

Nutrient deficiency is one of the major effects of waterlogging on plants, resulting in reduced photosynthesis and net carbon fixation ultimately leading to a reduction in growth and therefore yield (Bange et al., 2004). Application of essential nutrients will assist in mitigating the negative effects of abiotic stresses like waterlogging leading to increased productivity (Noreen et al., 2018). The use of enhanced-efficiency N fertilizers such as slow-release or controlled-release (SR/CR) fertilizers (Shaviv, 2001; Varadachari and Goertz, 2010) play an important role in improving plant growth and development under waterlogged conditions (Dinnes et al., 2002). Slow-release fertilizer can release nitrogen over a prolonged period during crop growth, thus maximize nitrogen-use efficiency (NUE) by synchronizing nitrogen release according to the crop demand (Lubkowski and Grzmil, 2007; Trenkel, 2010). Several studies (Ashraf et al., 2011; Habibzadeh et al., 2012; Najeeb et al., 2015) suggested that exogenously applied fertilizers could be effective if the nutrient ions enter into the root architecture, consequently, plants are able to recover from the injury caused by waterlogging. Application of fertilizer diminishes the effects of waterlogging of barley (Pang et al., 2007b), wheat (Kaur et al., 2017; Pereira et al., 2017; Zheng et al., 2017), maize (Rao et al., 2002), corn (Kaur et al., 2018), cotton (Guo et al., 2010; Wu et al., 2012; Li et al., 2013) and canola (Habibzadeh et al., 2012). In Australia, studies under both controlled-environments and field conditions have shown that additional CR urea application can mitigate waterlogging effects (Allen et al., 2010; Najeeb et al., 2015) of wheat and increase growth (Kisaakye et al., 2017) and grain yield by approximately 20% (Robertson et al., 2009). Similar findings reported by Mondal et al. (2018) and Swarup and Sharma (1993) showed that increased rates of top-dressed urea significantly increased wheat grain yield on flooded sodic soils in India. Likewise, the use of polyolefin-coated urea (a controlled-release fertilizer) resulted in a total N recovery of 66% in flood irrigated barley grown in north eastern Colorado, United States (Shoji et al., 2001). Fertilizer application also increases canopy duration and accelerates the production of photo-assimilates translocated to the grain compared with the straw thus increasing the harvest index (Kisaakye et al., 2015, 2017).

Potassium fertilizer has also been reported to ameliorate the detrimental effects of waterlogging in several crops including sugarcane (Sudama et al., 1998), rapeseed (Cong et al., 2009) and cotton (Ashraf et al., 2011). Exogenous application of various phosphorus (P) sources such as dairy cow manure (DCM) and meat and bone meal (MBM) is effective for producing optimum yields in P-deficient conditions during a wet growing season (Ylivainio et al., 2008, 2018). Application of farmyard manure also significantly increased grain Fe, Zn, Cu concentration of paddy under flooded conditions (Masunaga and Marques Fong, 2018). Similarly, foliar application of boron has been reported to increase overall plant growth and alleviate deleterious effect of waterlogging of maize (Sayed, 1998).

The use of fertilizers to alleviate waterlogging damage in broadacre cropping, even with high value crops, has been limited by lack of research and availability of information on their potential use in improving crop performance under waterlogged conditions (Lubkowski and Grzmil, 2007; Trenkel, 2010). Appropriate application methods, nutrient types, timing and rate should be considered to avoid the negative effect of tissue toxicities (e.g., manganese) (Silva et al., 2017; Huang et al., 2018) and nutrient imbalance on soil ecology (Rochester et al., 2001; Jackson and Ricard, 2003). The ability to predict waterlogging events (variable seasons) and therefore the crops’ nitrogen demand also limits the effectiveness of SR/CR fertilizers and therefore raises the question of whether highly available N applications would be preferable when waterlogging limits growth (Lubkowski and Grzmil, 2007; Trenkel, 2010). Robertson et al. (2007) suggested that pre-waterlogging application of N fertilizer might not be effective on wheat at the tillering stage. Application of nitrogen fertilizer during or immediately following waterlogging was less effective than pre-waterlogging due to inefficient nutrient ion absorption capacity of impaired roots, high leaching risks in the wet soils and at the late growth phase additional fertilizer applied could cause excessive vegetative growth and harvesting problems of cotton plant (Najeeb et al., 2015). Therefore, this strategy has limitations on a large-scale as the damaging effects of waterlogging can only be partially alleviated by the addition of fertilizers because of the reduced capability of roots to absorb nutrients (Trought and Drew, 1980; Kisaakye et al., 2015, 2017). For example, a drop in root membrane potential by 60 mV, often observed under hypoxic conditions (Gill et al., 2018) will require a 10-fold increase in cation (e.g., K^+^ or NH_4_^+^) concentration in the rhizosphere, to enable thermodynamically passive uptake (Gill et al., 2018). This approach is difficult to justify based on cost efficiency.

### Plant Growth Regulators

Plant growth regulators may mitigate waterlogging damage of plants by applying at the appropriate growth stage (Nguyen H.C. et al., 2018; Ren et al., 2018; Wu H. et al., 2018). The application of PGRs such as auxins and cytokinins has been reported to improve plant growth under waterlogged conditions (Pang et al., 2007b; Ren et al., 2016). The two hormones act in concert to promote stomatal conductance and photosynthetic capacity of waterlogged plants (Drew et al., 1979). Synthetic auxin 1-naphthaleneacetic acid (1-NAA), was reported to promote the growth of adventitious roots in waterlogged barley plants (Pang et al., 2007b) and; exogenous application of a cytokinin, 6-benzyladenine (6-BA) can alleviate waterlogging injuries and increase yield of maize (Ren et al., 2016, 2018). Pre-waterlogging foliar application of ABA increased tolerance to successive waterlogging-induced injury in cotton plant by improving photosynthesis of leaf (Pandey et al., 2002; Kim et al., 2018). Triazoles are known as fungitoxic and also have plant-growth regulatory effects and protect plants against various stresses (Leul and Zhou, 1998; Rademacher, 2015). For example, paclobutrazol mitigates waterlogging induced damage in canola and sweet potato plants (Lin et al., 2006). Uniconazole can also increase the chlorophyll content and the activity of antioxidant enzymes in canola (Leul and Zhou, 1999). Under waterlogging condition, the application of tricyclazole [5-methyl-1,2,4-triazole(3,4-b) benzothiazole] also mitigates the damage in plants (Habibzadeh et al., 2013). However, due to inconsistent results there has been little commercial use of PGRs to alleviate waterlogging damage.

### Combined Application of Fertilizer and Growth Regulators

Combined application of fertilizers and growth regulators can provide another option for ameliorating detrimental effects of waterlogging in crops, with the fertilizers acting as a nutrient supplier, while the PGRs assist with recovery from physiological injury (Li et al., 2013). 1% urea + 0.5% potassium chloride and growth regulators [brassin (0.02 mg/L) + diethyl aminoethyl hexanoate (10 mg/L)] improved growth and yield of waterlogged cotton (Li et al., 2013). Both foliar nutrient and PGRs application provide opportunities for future research.

### Use of Anti-ethylene Agents

Plant hypoxia-induced growth and yield losses could be the consequence of increased accumulation of ethylene (Shabala, 2011; Najeeb et al., 2018). Use of anti-ethylene agents such as 1-methylcyclopropene (1-MCP), amino ethoxyvinyl glycine (AVG), 1-aminocyclopropane-1-carboxylic acid (ACC), amino ethoxyacetic acid (AOA), silver and cobalt ions have been reported to inhibit the synthesis or accumulation of ethylene through blocking the biosynthetic pathway (Najeeb et al., 2017; Vwioko et al., 2017) of ethylene (McDaniel and Binder, 2012). Application of 1-MCP and AVG has been shown to diminish crop loss induced by ethylene accumulation (Kawakami et al., 2010; Najeeb et al., 2018). Brito et al. (2013) reported a positive effect of 1-MCP and AVG on cotton seed and lint yield. They determined that the initial reproductive phase is the best time for AVG application for improving cotton yield under waterlogging condition. In cotton, waterlogging prompts ethylene accumulation leading to young fruit abscission (Najeeb et al., 2017, 2018). During waterlogging conditions, an inverse link between ethylene production and cotton yield has also been found, therefore the application of AVG can regulate ethylene production and increase both photosynthesis and fruit retention of cotton (Bange et al., 2010; Najeeb et al., 2017). Likewise, the positive effect of 1-MCP has been studied on hypoxia cotton plants, where it also blocked ethylene action and enhanced physiological processes, such as antioxidant enzyme activity and stomatal resistance (Kawakami et al., 2010). Utilizing an ethylene-insensitive cotton mutant (eliminating ethylene sensitivity) may be another option for waterlogged areas, where the mutant plant showed a remarkably improved yield of cotton (Najeeb et al., 2017). There is further research required to fully understand the ethylene mediated pathways in other crops such as grains.

### Pretreatment With Hydrogen Peroxide

Pretreatment of crops with an agent may be an effective way to increase tolerance to different stresses (Jisha et al., 2013). For example, pretreatment with H_2_O_2_ can protect crops from oxidative damage caused by waterlogging, high light intensity, low temperature, salt stress, drought and exposure to heavy metals (Gechev et al., 2002; Ishibashi et al., 2011; Rajaeian and Ehsanpour, 2015; Savvides et al., 2016; Andrade et al., 2018). Priming seeds with H_2_O_2_ generated seedlings exhibiting elevated activity of antioxidant enzymes, low H_2_O_2_ and O_2_^-.^ content, and low cell membrane damage under waterlogged conditions (Andrade et al., 2018). H_2_O_2_ pre-treatment also resulted in increases in net photosynthetic rate and photosynthetic pigments, root volume, high biomass accumulation, and stem diameter (Andrade et al., 2018). Despite much research being conducted on priming with agents against biotic and abiotic stresses (Mustafa et al., 2017; Ashraf et al., 2018; Lal et al., 2018), pre-treatment with H_2_O_2_ tolerant to waterlogging still in its infancy.

### Use of Tolerant Species and Varieties

One of the key economical approaches for reducing the loss caused by waterlogging is to introduce waterlogging tolerance into existing plant varieties (Zhou, 2010; Tewari and Mishra, 2018; Wani et al., 2018). Genetic differences exist for tolerance to waterlogging in different crops (Setter and Waters, 2003) which include barley (Takeda and Fukuyama, 1986; Qiu, 1991; Pang et al., 2004; Xiao et al., 2007; Zhou et al., 2007; Huang et al., 2015; Zhang et al., 2015; Romina et al., 2018) and wheat (Davies and Hillman, 1988; Gardner and Flood, 1993; Huang et al., 1994; Herzog et al., 2016; Nguyen T.N. et al., 2018; Wu X. et al., 2018). However, waterlogging tolerance is a complex trait which is controlled by many different mechanisms, such as aerenchyma formation in roots (Zhang et al., 2015; Luan et al., 2018; Pujol and Wissuwa, 2018) under waterlogging stress, tolerance to secondary metabolites (Pang et al., 2006), ion toxicities (Huang et al., 2018), the maintenance of membrane potential (Gill et al., 2018) and control of ROS production under stress, with many QTL being reported to control these traits (Li et al., 2008; Zhou, 2011; Zhang et al., 2017; Huang et al., 2018; Gill et al., 2018). The success of a breeding program relies on the discovery of genes and linked markers to various tolerance mechanisms, which enable breeders to pyramid tolerance genes.

## Summary and Recommendations

**FIGURE 10:**
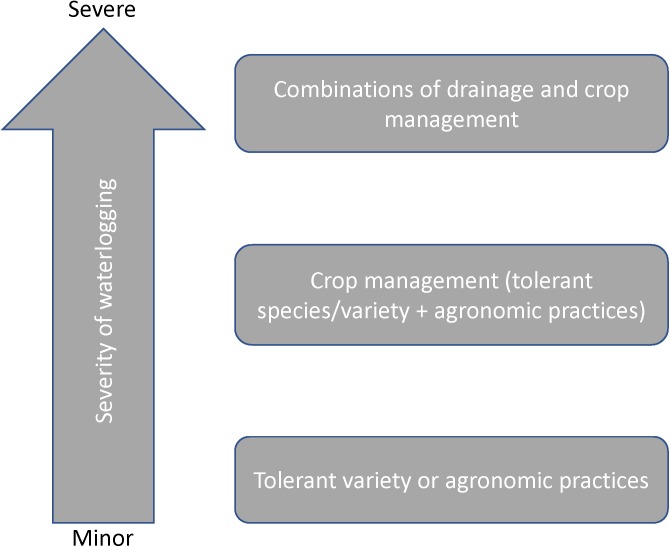
Recommendation of soil and crop management based on different waterlogging severity.

Many soil and crop management practices have been employed to alleviate waterlogging in crop production systems as summarized in Table 1. For severe waterlogging, combinations of drainage and crop management will be the foremost step (Figure 10). For minor waterlogging, choosing tolerant varieties or applying appropriate agronomic practices can be effective.

**Table 1:** Summary of advantages and disadvantages of different soil and crop management practices.

Soil and crop management practices	Advantages (in addition to reducing waterlogging)	Disadvantages	Reference
Surface drainage	Both installation and maintenance are simplest and cheapest	Open drains with less cropping area; needs periodic maintenance	Food and Agriculture Organization [FAO], 2002; Ritzema et al., 2008; Ayars and Evans, 2015; Palla et al., 2018
Raised bed system	Improvements in soil structure	Efficiency depends on height of water table; poorer weed control in furrows; cost of modifying machinery; less cropping area	Bakker et al., 2005b, 2007; Roth et al., 2005; Zhang, 2005; Acuña et al., 2011; Gibson, 2014
Pipe drains	Well tested method for severe waterlogging	Needs outfall and periodic maintenance; cost of installation is high	Tanji, 1990; Food and Agriculture Organization [FAO], 2002; Filipović et al., 2014; Teixeira et al., 2018
Vertical drainage	Well tested method for severe waterlogging	Maintenance and operational costs are higher than for horizontal pipe drainage systems	Christen et al., 2001; Food and Agriculture Organization [FAO], 2002; Kijne, 2006; Prathapar et al., 2018
Mole drains	Well tested method; cheaper than other underground drainage	Needs periodic maintenance; will not maintain integrity in dispersive soils	Tuohy et al., 2016, 2018; Dhakad et al., 2018
Controlled traffic farming (CTF)	Reduced soil compaction, erosion, tillage costs, water and nutrient losses	Variable results with different conditions, such as different crops, soil types and tillage	Zhang, 2005; Chamen et al., 2006; Guenette and Hernandez-Ramirez, 2018; Thomsen et al., 2018; Bennett et al., 2019
Strategic deep tillage and subsoil manuring	Decreases soil strength resulting in deeper and denser rooting	SDT with no added amendment is often short-term nature, less effective in hostile sub-soils, such as acidity, sodicity or subsoil salinity	Gajri et al., 1994; Bakker et al., 2007; Roper et al., 2015
Early sowing and vigorous crop	Use of existing soil water provides a buffer; avoids terminal waterlogging events	Minor benefit with severe waterlogging	Stapper and Harris, 1989; Setter and Waters, 2003; Bassu et al., 2009; Ploschuk et al., 2018; Sundgren et al., 2018; Wollmer et al., 2018
Bio-drainage	Tried and tested at many locations with success	Needs proper plantation techniques, expertise, thinning, pruning, and harvesting	Kapoor, 2000; Food and Agriculture Organization [FAO], 2002; Heuperman and Kapoor, 2003; Dash et al., 2005; Lin et al., 2011; Lerch et al., 2017; Muñoz-Carpena et al., 2018; Sarkar et al., 2018; Singh and Lal, 2018;
Nutrient application, in particular, N	Improving plant growth and development	Appropriate methods, nutrient types, timing and rate should be considered for large-scale application	Rao et al., 2002; Pang et al., 2007b; Guo et al., 2010; Ashraf et al., 2011; Habibzadeh et al., 2012; Wu et al., 2012; Li et al., 2013; Najeeb et al., 2015; Kaur et al., 2017, 2018; Pereira et al., 2017; Zheng et al., 2017
Plant growth regulators	Promote stomatal conductance and photosynthetic capacity of waterlogged plants	Appropriate methods, timing and rate should be considered for large-scale application; unproven in broad scale agriculture	Drew et al., 1979; Lin et al., 2006; Habibzadeh et al., 2013; Ren et al., 2016, 2018
Use of anti-ethylene agents	Increase both photosynthesis and fruit retention; diminish crop loss induced by ethylene accumulation	Untested in broad scale agriculture	Kawakami et al., 2010; Shabala, 2011; Najeeb et al., 2018
Pretreatment with hydrogen peroxide	Protect crops from oxidative damage caused by waterlogging	Untested in broad scale agriculture	Gechev et al., 2002; Ishibashi et al., 2011; Rajaeian and Ehsanpour, 2015; Savvides et al., 2016; Andrade et al., 2018
Tolerant species and varieties	Cost effective for farmers	The introduction of waterlogging tolerance into existing plant varieties is time consuming and complex	Davies and Hillman, 1988; Gardner and Flood, 1993; Zhou et al., 2007; Gill et al., 2018; Huang et al., 2018


There are still significant knowledge gaps in our understanding of the advantages or disadvantages of relevant management measures under different soil types or different crops, management of other macro- and micronutrients; and the genetic basis of plants’ adaptation to hypoxia and elemental toxicities in waterlogged soils. While many tolerance mechanisms and related quantitative trait loci (QTL) have been reported, most of them are focused around oxygen availability and largely ignore other constraints imposed by waterlogged soils.

For improved mitigation strategies, further research should be focused on the following aspects:

- Comparison of the cost/benefit analyses of different drainage strategies;
- Understanding the mechanisms of nutrient loss during waterlogging and quantifying the benefits of nutrient application;
- Increasing soil profile de-watering through soil improvement and agronomic strategies;
- Increased specificity of the interaction between different management practices and environment (soil types, severity of waterlogging, etc.) as well as among management practices.
- Discovering new (non-oxygen-associated) QTLs; the effectiveness of these mechanisms/QTL (and combined) on improving waterlogging tolerance in paddocks with soils with multiple constraints; the effect of these QTL on other agronomic, yield and quality traits, as well as management packages for varieties with diverse waterlogging tolerance genes.

## Author Contributions

SM and GP prepared the draft. GD supervised the project. GP, GD, BF, SS, and MZ reviewed and revised the manuscript.

## Conflict of Interest Statement

The authors declare that the research was conducted in the absence of any commercial or financial relationships that could be construed as a potential conflict of interest.
